# The Role of Circular RNAs in the Carcinogenesis of Bladder Cancer

**DOI:** 10.3389/fonc.2022.801842

**Published:** 2022-02-28

**Authors:** Soudeh Ghafouri-Fard, Sajad Najafi, Bashdar Mahmud Hussen, Abbas Basiri, Hazha Jamal Hidayat, Mohammad Taheri, Fariborz Rashnoo

**Affiliations:** ^1^ Department of Medical Genetics, School of Medicine, Shahid Beheshti University of Medical Sciences, Tehran, Iran; ^2^ Student Research Committee, Department of Medical Biotechnology, School of Advanced Technologies in Medicine, Shahid Beheshti University of Medical Sciences, Tehran, Iran; ^3^ Department of Pharmacognosy, College of Pharmacy, Hawler Medical University, Erbil, Iraq; ^4^ Urology and Nephrology Research Center, Shahid Beheshti University of Medical Sciences, Tehran, Iran; ^5^ Department of Biology, College of Education, Salahaddin University-Erbil, Erbil, Iraq; ^6^ Institute of Human Genetics, Jena University Hospital, Jena, Germany; ^7^ Skull Base Research Center, Loghman Hakim Hospital, Shahid Beheshti University of Medical Sciences, Tehran, Iran

**Keywords:** bladder cancer, ncRNAs, circRNAs, expression, biomarker

## Abstract

Circular RNAs (circRNAs) are a group of transcripts with enclosed configurations which can regulate gene expression. These transcripts have important roles in normal development and in the pathogenesis of disorders. Recent evidence has supported involvement of circRNAs in the development of bladder cancer. Several circRNAs such as circ_0058063, hsa-circRNA-403658, circPDSS1, circCASC15, circRNA-MYLK, and circRNA_103809 have been upregulated in bladder cancer samples. On the other hand, hsa_circ_0137606, BCRC-3, circFUT8, hsa_circ_001598, circSLC8A1, hsa_circ_0077837, hsa_circ_0004826, and circACVR2A are among downregulated circRNAs in bladder cancer. Numerous circRNAs have diagnostic or prognostic value in bladder cancer. In this review, we aim to outline the latest findings about the role of circRNAs in bladder cancer and introduce circRNAs for further investigations as therapeutic targets.

## Introduction

Non-coding RNAs (ncRNAs) comprise several groups of RNA transcripts whose no protein is known to be encoded and thus considered as junk; however, they constitute a majority of expressed RNAs compared to protein-coding transcripts (1). Circular RNAs (circRNAs) are a distinct class of ncRNAs in eukaryotic cells which have been identified *via* electron microscopy in 1979 for the first time (2). Unlike coding-RNAs, circRNAs lack the 5′ cap and 3′ polyadenylated tail and do not mainly encode any protein; therefore, no primary function has been described for them (3). However, peptide-coding circRNAs have also been recognized. Some findings have revealed developmental, pathogenic, and especially regulatory roles for circRNAs. As their name suggests, a close circular loop in circRNAs is formed by covalent linkages between the 5′ and 3′ ends of their transcripts. CircRNAs compared to their linear counterparts show higher stability against degrading agents like RNase R due to closed ends (4) but are found in lower quantities within the animal cells (5), although higher abundance is reported for some circRNAs (6). Their sequence is evolutionarily conserved, indicating selective pressure for them (6). CircRNAs also show specific cell and tissue tendencies (7). Their elevated levels in several diseases demonstrate their potentials as diagnostic biomarkers and also therapeutic potentials especially in cancers (8). Today, due to development of advanced technologies like high-throughput RNA sequencing (RNA seq) and *in situ* experiments, a huge number of circRNAs have been recognized in animal cells (2). Moreover, their regulatory roles in gene expression and pathogenesis of disorders have been recognized. Similar to other regulatory non-coding RNAs, regulatory functions of circRNAs are suggested to be exerted through modulating gene expression at different levels. Based on the gene region, circRNAs can be divided into three types: those originating from exons (exonic circRNAs), introns (intronic circRNAs), or exon–intron junctions (exon–intron circRNAs) (9). Exonic circRNAs have been found in higher concentrations in cytoplasm compared to the nucleus showing capability of sponging microRNAs (miRNAs) and so can positively affect the expression of target genes leading to their overexpression. Unlike the first type, the other types are more concentrated in the cell nucleus and thus regulate gene expression at the primary steps of transcriptional and posttranscriptional levels (10, 11). CircRNAs have been widely detected in different cells, tissues, and organisms and also during various stages of organism development, playing a role in controlling cell growth and stress (12). Through their regulatory mechanisms in the cell cycle, circRNAs have been found to apply surveillance on eukaryotic cell proliferation and homeostasis. Consistent with these findings, dysregulation of circRNAs has been reported in a vast number of proliferative disorders like different tumors. Bladder cancer (BCa) is an example in which the role of aberrantly expressed circRNAs in tumor development and progression has been studied. Similar to other malignancies, response to treatment in BCa requires early diagnosis which also guarantees better prognosis for the patients. CircRNAs not only have acted as potential biomarkers for BCa with promising characteristics in diagnosis and prediction of prognosis in BCa patients but also have been suggested as therapeutical targets in fighting against malignancy. In this review, we aim to outline the latest findings about the role of circRNAs in BCa.

## CircRNAs in BCa

### Upregulated CircRNAs in BCa

In expression analyses *via* high-throughput technologies like microarray and sequencing and also in quantitative PCR studies, a number of circRNAs have been found to be upregulated in samples taken from patients with BCa compared to healthy controls. These kinds of circRNAs are suggested as oncogenes with carcinogenic roles. Accordingly, their overexpressed levels have been shown to promote tumor cell proliferation and invasion in cell studies and also *in vivo* experiments, while their downregulation or knockdown reverses these effects.

Microarray analysis provides a possibility to screen a large number of aberrantly expressed circRNA candidates in a single platform. CircRNA_0058063 is an example which was reported recently by Liang et al. (13) as an upregulated circRNA in cancerous tissues of BCa patients compared to adjacent normal tissues. Microarray results revealed 312 aberrantly expressed circRNAs including 195 upregulated and 117 downregulated ones. CircRNA _0058063 showed a significantly increased expression in both BCa cell lines and tissues. A reverse correlation was seen between circRNA _0058063 expression level and overall survival (OS) in patients. Consistent with expectations, circRNA _0058063 knockdown suppressed tumor cell proliferation and metastasis in BCa BIU-87 cell lines. CircRNA_0058063 was found to act as a miRNA sponge to decrease the expression level of miR-486-3p by making interaction *via* a complementary sequence and induction of silencing. miR-486-3p inhibits the expression of the FOXP4 transcription factor which promotes tumorigenicity in various cancers.

Li et al. (14) using high-throughput sequencing found a number of upregulated RNA transcripts including long non-coding RNAs (lncRNAs), protein-coding mRNAs, and 34 circRNAs in 20 BCa tissues compared with a matched number of adjacent normal bladder tissues in addition to another set of transcripts which were downregulated. In GO and KEGG pathway enrichment analyses, dysregulated RNAs were associated with several signaling pathways controlling different critical cellular processes particularly DNA replication and cell cycle which play a role in the pathogenesis of BCa. In addition, 3 circRNAs including circPGM5 and circKIAA1462 were validated by qPCR. CircRNA PGM5 was demonstrated in the competing endogenous RNA (ceRNA) network to possess recognition sites for miRNAs associated with BCa along with lncRNA MIR194-2HG and AATBC.

Also, robust next-generation sequencing (NGS) technique and confirmatory qRT-PCR have been used to screen dysregulated circRNAs in BCa samples (15). A significant differential expression of a single circRNA is assessed in BCa tissues relative to adjacent normal tissues *via* quantitative reverse transcription polymerase chain reaction (qRT-PCR; also known as real-time RT-PCR). An increasing number of circRNAs have been reported in separate studies to be aberrantly expressed in BCa tissues or in serum or urine samples of patients; thus, these circRNAs have been suggested as potential diagnostic and prognostic biomarkers for BCa patients (16). Hsa-circRNA-403658 is a good instance for a series of circRNAs found to be upregulated in BCa tissues *via* the latter method. Wei *et al.* (17) demonstrated a differential expression of a number of circRNAs in BCa SW780, 5637, T24, J82, and RT4 cell lines cultured in hypoxic conditions in comparison with CCC-HB-2 healthy bladder epithelial cells using circRNA microarray. Hsa-circRNA-403658 was one of these circRNAs which showed the highest level of increased expression in further evaluation by qRT-PCR assay. Clinical samples of patients with BCa in which higher levels of hsa-circRNA-403658 were seen showed poorer prognosis, larger tumor size, increased metastasis, and higher clinicopathological stage (TNM staging) compared to patients with lower hsa-circRNA-403658 expression levels. As expected, hsa-circRNA-403658 knockdown using specific silencing RNA (siRNA) inhibited circRNA tumorigenic effects. Furthermore, *in vitro* and *in vivo* studies showed that hsa-circRNA-403658 controls the anaerobic glycolysis in hypoxic culture *via* enhancing the promotor activity and consequently positive regulation of lactate dehydrogenase A (LDHA) expression.

**Figure 1:**
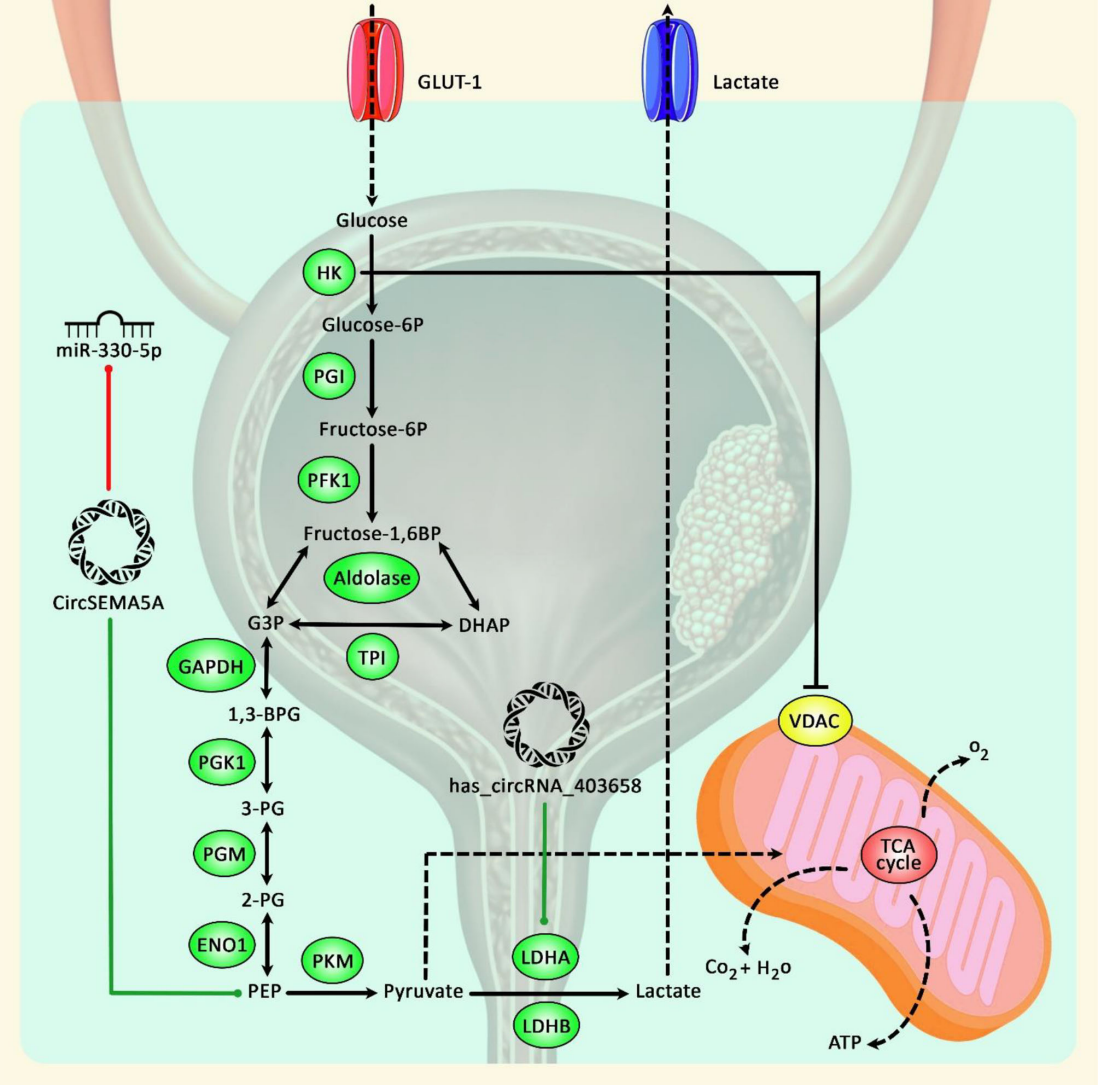
A schematic diagram of the role of circRNAs in promoting bladder cancer progression *via* enhancing glycolysis. Accumulating findings have suggested that upregulation of key glycolysis proteins could play a crucial role in cancer development. As an illustration, a previous study has authenticated that has_circRNA_403658 through upregulation of LDHA-mediated aerobic glycolysis could have a significant part in enhancing bladder cancer cell growth (17). In addition, another research has detected that circSEMA5A *via* sponging miR-330-5p could upregulate the expression level of ENO1, thereby elevating proliferation, invasion, angiogenesis, and glycolysis of bladder cancer cells by facilitating the activation of Akt and β-catenin signaling cascades (21). Green arrows indicate the upregulation of target genes by circRNAs, and red arrows depict inhibitory effects.

Computational studies and network analyses using bioinformatics methods have also facilitated the detection of differentially expressed circRNAs and their pathological roles and potential application as novel biomarkers for BCa (18–20), suggesting their application in early diagnosis and prediction of prognosis as well as their therapeutic potentials. **Figure 1** demonstrates the role of circRNAs in modulating bladder cancer development *via* promoting glycolysis.

**Table 1** outlines the most important circRNAs with an elevated expression level in the BCa cell line and also in patients’ samples.

**Table 1:** Upregulated circRNAs in BCa.

CircRNA (other terms)	Clinical cases	Cell lines	Target genes/regulators/sponged miRNAs	Affected signaling pathway/process	Findings on overexpressed or silenced circRNA in BCa cellular experiments	Ref. (s)
Circ_0058063	94 BCa and matched NATs	HEK293 and BIU-87, 5637, and RT-112	miR-486-3p/FOXP4 axis miR-145‐5p/CDK6 axis	–	Enhanced metastasis, correlation with higher disease stage	(13, 22)
Has_circRNA_403658	123 BCa patient tissues and matched NATs	CCC-HB-2 normal bladder epithelial cells and BCa SW780, 5637, T24, J82, and RT4	LDHA	Glycolysis	Poorer prognosis, larger tumor size, increased metastasis, and higher clinicopathological stage	(17)
CircPDSS1	72 patient tissues and their NATs	HT-1197 and UMUC3	miR-16	–	Increased tumor cell proliferation, migration, and invasion	(23)
CircCASC15 (hsa_circ_0075828)	67 patient tissues and control para-carcinoma tissues	5637, J82, UM-UC-3, T24, and SW780 human BC cell lines and SV-HUC-1 human uroepithelial cell line	miR-1224-5p and CREB1	–	Accelerated cell proliferation	(24)
CircRNA-MYLK	32 patient tissues and control para-carcinoma tissues	EJ, T24, 5673, and BIU-87 BCa cell lines and 293T human embryonic kidney cells	miR-29a, VEGFA	Ras/ERK	Promoted cell proliferation, migration, and epithelial–mesenchymal transition (EMT) *in vitro* and enhanced angiogenesis and metastasis in xenografts	(25)
Hsa_circ_0001361	69 patient tissues and healthy bladder epithelial tissues as matched controls	EJ, UMUC3, RT4, 5637 human BCa cell lines, and SV-HUC-1 uroepithelial cell line	miR-491-5p, MMP9	–	Facilitated *in vitro* and *in vivo* invasion and metastasis	(26)
Circ-VANGL1	87 BCa patient tissues and 37 NATs	T24, 253J, UMUC-3, J82, 5637, and EJ BCa cell lines and	miR-605‐3p/VANGL1 axis	–	Promoted *in vitro* cell proliferation, migration, and invasion and *in vivo* BCa propagation	(27)
CircTCF25	4 pairs of BCa tissues and matched NATs in microarray analysis and 40 pairs for qRT-PCR validation	T24 and EJ BCa cell lines	miR-103a-3p/miR-107/CDK6 axis	–	Promoted *in vitro* and *vivo* proliferation and migration	(28)
Hsa_circ_0137439	Urine samples of 10 BCa patients and 10 healthy controls in microarray analysis and 116 BCa samples plus 30 healthy controls in qRT-PCR validation	T24 and 5637 human BCa cell lines	miR-142-5p/MTDH axis	–	Promoted BCa cell proliferation and metastasis	(29)
CircPTK2 (has-circ-0003221)	40 BCa tissues and matched para-carcinoma NATs	T24 and 5637 BCa cell lines	–	–	Promoted BCa cell proliferation and migration	(30)
CircCEP128	40 BCa tissues and matched NATs	293T, J82 and T24 BCa cell lines and SV-HUC-1	miR-145-5p/*MYD88* axis	MAPK	CircCEP128 silencing inhibited cell viability and mobility and stimulated apoptosis	(31)
Circ0001429	20 BCa tissues and matched NATs	T24 and 5637 SV-HUC-1 and BIU-87	miR-205-5p/VEGFA axis	–	Enhanced propagation, migration, and invasion, and inhibited apoptosis	(32)
CircRGNEF	90 BCa patient tissues and matched NATs	J82, EJ, T24, TCC, UM-UC-3, and RT-4 BCa cell lines and SV-HUC	miR-548/KIF2C axis	–	Promoted BCa cell proliferation, migration, and invasion	(33)
CircGPRC5a (hsa_circ_02838)	20 early BCa, 40 advanced BCa samples, and 60 NATs	Bladder CSCs	circGprc5a-peptide/Gprc5a axis	–	Promoted self-renewal and invasion of bladder CSCs	(34)
Hsa_circ_0000144	69 BCa patient tissues and 21 matched NATs	T24, EJ, UMUC3, RT4, and 253J BCa cell lines and SV-HUC-1 cell line	miR-217/RUNX2 axis	–	Promoted *in vitro* and *in vivo* BCa cell proliferation and invasion	(35)
CircINTS4	40 BCa samples and 40 NATs	RT4, SW780, J82, 5637, T24, UMUC3 BCa cell lines, and SV-HUC	miR-146b/CARMA3 axis	NF-kB ↑ P38 MAPK ↓	Increased *in vitro* and *in vivo* BCa tumorigenicity	(36)
CircZFR	104 BCa samples and 40 NATs	UMUC3, T24, J82, 5637, SW780, EJ and BIU87 BCa cell lines, and CCC-HB-2 cells	miR-377/ZEB2 axis	–	Silencing showed inhibition of cell proliferation, migration, and invasion	(37)
CircASXl1 (hsa_circ_0001136)	61 BCa samples and 40 NATs	–	–	–	Correlated with worse clinicopathological features in BCa patients and lower OS	(38)
Hsa_circ_0068871	32 BCa samples and 40 NATs	T24, UMUC3, EJ and J82 BCa cell lines and SV-HUC-1	miR-181a-5p/FGFR3 axis	–	Promoted tumor cell growth	(39)
Circ_102336	64 BCa samples and 40 NATs	T24 and 5637 BCa cell lines and SV-HUC-1 and HEK-293 T cells	miR-515-5p	–	Promoted cell growth Enhanced drug sensitivity in circ_102336 knockdown	(40)
Hsa_circ_0068307	30 BCa samples and 40 NATs	EJ, T24, RT-4 and UM-UC-3 BCa cell lines	miR-147/c-Myc axis	–	Hsa_circ_0068307 knockdown inhibited BCa *in vitro* cell proliferation and migration and *in vivo* xenografts	(41)
Circ_0006332	32 BCa samples and 40 NATs	5637, T24, J82, UM-UC-3, TSCCUP, and SV-HUC-1 BCa cell lines	miR-143/MYBL2 axis	–	Promoted *in vivo* cancer growth Circ_0006332 knockdown suppressed BCa cell proliferation and invasion	(42)
CircRNA-0071196	80 BCa tissues and 30 para-carcinoma tissues	5637 BCa cell line	miR-19b-3p/CIT axis	–	CircRNA-0071196 silencing decreased BCa cell proliferation and migration	(43)
circZNF139	–	UC3 and 5637 cells BCa cell lines	–	PI3K/AKT	ZNF139/circZNF139 promoted BCa cell proliferation, migration, and invasion	(44)
CircDOCK1	32 BCa samples and 32 NATs	BIU-87, EJ-m3, T24 and 5673 BCa cell lines and SV-HUC-1 cells	hsa-miR-132-3p/Sox5 axis	–	CircDOCK1 silencing suppressed BCa cell progression *in vitro* and xenograft growth *in vivo*	(45)
CircKIF4A (hsa_circ_0007255)	50 BCa samples and 32 NATs	5637, RT-112, and BIU-87 BCa cell lines and HEK293T cells	miR-375/1231/NOTCH2 axis	–	Promoted BCa *in vitro* cell proliferation and metastasis	(46)
Hsa_circ_0001944	90 BCa samples and 32 NATs	5637, UM-UC-3, T24, and RT-4 BCa cell lines and SV-HUC-1 cells	miR-548/PROK2 axis	–	Silencing suppressed BCa cell proliferation and invasion *in vitro* and *in vivo*	(47)
CircPRMT5	119 BCa samples and 32 NATs	T24, TCC-SUP, 5637, and UM-UC-3 BCa cell lines, and SV-HUC-1 cells	miR-30c/SNAIL1/E-cadherin axis	–	Silencing decreased BCa cell migration, invasion *in vitro*, and metastasis *in vivo* Overexpression enhanced BCa cells EMT	(48)
CircGLIS3 (hsa_circ_0002874)	48 BCa samples and 32 NATs	T24, UM-UC-3 BCa cell lines and SV-HUC-1 cells	miR-1273f/SKP1/cyclin D1 axis		Silencing inhibited BCa cell proliferation, invasion, and migration *in vitro* and cell growth *in vivo* Upregulation promoted G0/G1 phase of cell cycle through miR-1273f/SKP1/Cyclin D1 axis	(49)
Hsa_circ_0041103	50 BCa samples and 32 NATs	T24, UM-UC-3, RT4, 5637 BCa cell lines, and SV-HUC-1 cells	miR-107/FOXK1 axis	–	Silencing inhibited BCa cell proliferation and metastasis	(50)
CircEHBP1	186 BCa samples and 32 NATs	UM-UC-3, T24, and 5637 BCa cell lines, and SV-HUC-1 cells	miR-130a-3p/TGFβR1/VEGF-D axis	TGF-β/SMAD	Promoted BCa lymph node metastasis *in vivo*	(51)
Circ_0000735	50 BCa samples and 32 NATs	5637, RT-112 and BIU-87 BCa cell lines, and SV-HUC-1 cells	miR-502-5p	–	Knockdown suppressed BCa cell proliferation and invasion *in vitro* and tumorigenesis *in vivo*	(52)
Circ_100984	20 BCa samples and 32 NATs	HT-1376, HTB9, 253J, BT-B, Biu-87 and 5637 BCa cell lines and SV-HUC-1 cells	miR-432-3p/c-Jun/YBX-1/β-catenin axis	Wnt	Knockdown inhibited BCa cell growth, invasion, metastasis, and EMT *in vivo* and *in vivo*	(53)
circRIMS1 (hsa_circ_0132246)	20 BCa samples and 32 NATs	J82, 5637, T24, EJ, and TCCSUP BCa cell lines and SV-HUC-1 and HEK-293 cells	miR-433-3p/CCAR1/c-Myc axis	–	Knockdown repressed BCa cell proliferation, invasion, and metastasis *in vivo* and tumor growth *in vivo*	(54)
CircSEMA5A	40 BCa samples and 32 NATs	T24, UM-UC-3, 5637, J82 BCa cell lines, and SV-HUC-1 cells	miR-330-5p/ENO1 axis	Glycolysis ↑	Promoted BCa cell proliferation, invasion, migration capabilities, and angiogenesis *in vivo*	(21)
CircRNA_100146 **(**hsa_circRNA_100146)	68 BCa samples and 32 NATs	J82, 5637, SW780, and T24 BCa cell lines, and HCV-29 cells	miR-149-5p/RNF2 axis	–	Promoted BCa cell proliferation, invasion, and migration and inhibited apoptosis	(55)
CircBC048201	30 BCa samples and 32 NATs	UM-UC-3 BCa cell lines and SV-HUC-1 cells	miR-1184/ITGA3 axis	–	Interference inhibited BCa cell proliferation, migration, and invasion	(56)
CircRNA_0071196	80 BCa samples and 30 matched para-carcinoma tissues	The 5637 human BCa cell line	miR-19b-3p/CIT axis	–	Knockdown repressed BCa cell proliferation and migration	(43)
Circ_0061140	42 BCa samples and corresponding NATs	T24, 253j, 5637, J82, RT4, UMUC3 BCa cell lines, and SV-HUC-1 cells	miR-1236	–	Circ_0061140 knockdown repressed BCa cell proliferation and invasion	(57)
Circ_001418	–	–	miR-1297/EphA2 axis	–	Enhanced BCa cell proliferation and invasion, and decreased apoptosis	(58)
Circ_0067934	54 BCa tissues and corresponding NATs	T24, RT4 and UMUC3 BCa cell lines, and SV-HUC-1 cells	miR-1304-Myc axis	–	Circ_0067934 silencing inhibited BCa cell proliferation, invasion, and migration *in vivo*	(59)
Hsa_circ_0017247	50 BCa tissues and corresponding NATs	UM-UC3, SW780, BIU, and J82 BCa cell lines	–	Wnt/β-catenin	Knockdown inhibited BCa cell growth and promoted apoptosis *in vitro* and repressed tumor growth *in vivo*	(60)
CircFNTA	41 BCa tissues and corresponding NATs	T24, J82, 5637, and UMUC3 BCa cell lines, and SV-HUC cells	miR-370-3p/FNTA axis	KRAS	Enhanced cell invasion and chemo-resistance to cisplatin in BCa cell lines CircFNTA knockdown repressed its tumorigenic effects	(61)
CircRIP2	58 BCa tissues and corresponding NATs	5637 and UM-UC-3 BCa cell lines	miR-1305	Tgf-β2/smad3	Increased BCa cell progression through stimulation of EMT	(62)
CircUVRAG	Experiment was conducted on 20 BALB/c nude mice	T24, EJ, J82, UM-UC-3, TCC, and RT-4 BCa cell lines, and SV-HUC cells	miR-223/FGFR2 axis	–	Knockdown repressed BCa cell proliferation and metastasis *in vitro* and *in vivo*	(63)
Circ-BPTF	72 BCa tissues and corresponding NATs	UM-UC-3 and T24 BCa cell lines	miR-31-5p/RAB27A axis	–	Increased *in vitro* and *in vivo* progression of BCa cells	(64)
Circ_0023642	–	J82 and UMUC3 BCa cell lines	miR-490-5p	ERα/circ_0023642/miR-490-5p/EGFR	ERα suppressed BCa cell invasion *in vitro* through downregulation of circ_0023642 *via* expressional modulation of UVRAG host gene and also repressed metastasis *in vivo*	(65)

↑, activation or increased level; ↓, inhibition or decreased level; NAT, normal adjacent tissue.

VEGFA, vascular endothelial growth factor A; MMP9, matrix metallopeptidase 9; MTDH, metadherin; CSCs, cancer stem cells; IGFIR, type 1 insulin-like growth factor receptor; ERα, estrogen receptor alpha.

### Downregulated CircRNAs in BCa

These kinds of circRNAs have been found to exhibit lower expression levels in BCa samples compared to normal adjacent tissues. They are suggested to play a role as tumor suppressors with biological functions controlling the critical cellular processes cell proliferation and extracellular matrix stability and so their downregulation in a set of studies has been shown to facilitate tumor cell proliferation, migration, and invasion. *In vivo* experiments have also demonstrated accelerated tumor progression in the presence of decreased expression of these circRNAs. Similar to the former circRNAs, these kinds have been linked with cell cycle regulation through different miRNA–protein axes, among which are some oncogenes or tumor-suppressor genes which are dysregulated. Bioinformatic analyses, RNA pull-down assays, and luciferase reporter assays have shown single or several miRNAs being sponged in close interaction with circRNAs. The expression of these miRNAs is mainly suppressed *via* upstream circRNAs, and they themselves regulate some actions through affecting downstream molecules. Yet, some miRNAs act upstream of circRNAs exerting a regulatory role on them. Downregulated circRNAs are mainly located in the cytoplasm, so it is suggested that they play their regulatory roles at posttranscriptional or translational steps.

For instance, circSLC8A1 is a circRNA which has been reported by Lu *et al.* (66) to be downregulated in BCa tissues compared to healthy adjacent tissues in a study of 70 patients diagnosed with BCa. They detected a number of aberrantly expressed circRNAs through RNA sequencing. qRT-PCR confirmed a decreased expression of circSLC8A1 in 81% (57/70) of total BCa tissues compared to their matched adjacent tissues. Expression assay in 6 BCa cell lines using qRT-PCR also showed a decreased expression of circSLC8A1 compared to SV-HUC-1 normal bladder cells.

**Figure 2:**
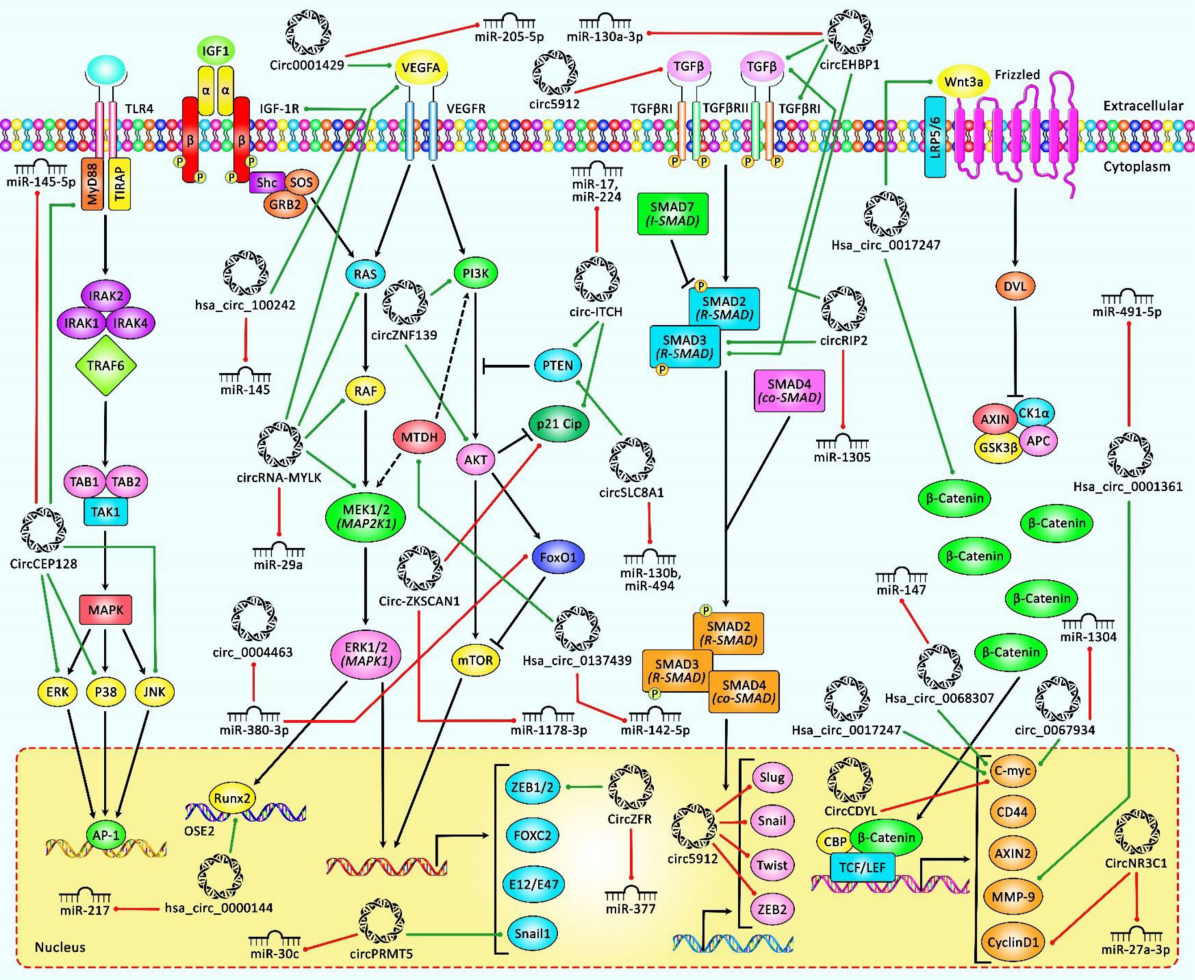
A schematic representation of the role of various circRNAs in human bladder cancer through modulating the PI3K/AKT, MAPK/ERK, TGFβ/SMAD3, and Wnt/β-catenin signaling pathways. According to the diagram, the upregulation or downregulation of several circRNAs could have a considerable role in bladder cancer development through modulation of miRNA levels. Green arrows indicate upregulation of target genes by circRNAs, and red arrows depict inhibition regulated by them. All the information regarding the role of these circRNAs in bladder cancer is shown in **Tables 1**, **2**.

*In vitro* analyses revealed suppression of tumorigenic impacts following circSLC8A1 overexpression in BCa cell lines. By using different prediction tools, it was demonstrated that circSLC8A1 potentially sponges 7 miRNAs, among which were miR-130b and miR-494 whose interactions with circSLC8A1 were confirmed by RNA pull-down assay and biotin labeling. Overexpression of both miRNAs was associated with oncogenesis in BCa cells. Furthermore, luciferase reporter assay and Western blotting analysis demonstrated that miRNAs can bind to the 3′ end of the phosphatase and tensin homolog (PTEN) tumor suppressor and inhibit its expression. Rescue experiments and immunohistochemistry (IHC) analysis showed that circSLC8A1 acts as a tumor suppressor *via* the miR-130b and miR-494/PTEN/PI3k/Akt signaling axis. **Table 2** summarizes the recent findings of tumorigenicity studies on downregulated circRNAs in BCa. **Figure 2** represents the role of several circRNAs in bladder cancer cells *via* regulating some key signaling cascades.

**Table 2:** Downregulated circRNAs in BCa.

circRNA (other terms)	Clinical cases	Cell lines	Target genes/regulators/sponged miRNAs	Affected signaling pathway/process	Findings on overexpressed or downregulated circRNA in BCa cellular experiments	Ref. (s)
CircBCRC-3	47 BCa patient tissues and matched NATs	BC and EJ BCa cell lines and SV-HUC-1 cells	miR-182-5p/p27 axis	–	Overexpression inhibited BCa cell growth *in vitro* and tumor progression *in vivo*	(67)
CircFUT8	145 BCa patient tissues and 50 matched NATs	T24 and UM-UC-3 BCa cell lines and SV-HUC-1 cells	miR-570-3p/KLF10 axis	–	Overexpression suppressed BCa cell migration and invasion *in vitro* and metastasis *in vivo*	(68)
BCRC4 (hsa_circ_001598)	24 BCa patient tissues and matched NATs	UMUC3 BCa cell lines and SV-HUC-1 cells	miR-101/EZH2 axis	–	Overexpression decreased BCa cell proliferation and also, increased apoptosis	(69)
CircSLC8A1	70 BCa patient tissues and matched NATs	5637, T24, J82, EJ, UMUC, and RT4 BCa cell lines, and SV-HUC-1 cells	miR-130b and miR-494/PTEN/PI3k/Akt signaling axis	PI3k/Akt	Overexpression decreased BCa cell migration and invasion *in vitro* CircSLC8A1 inhibited tumor progression *in vivo*	(66)
Hsa_circ_0077837 and Hsa_circ_0004826	70 BCa patient tissues and matched NATs	EJ, 5637, and T24 BCa cell lines and SV-HUC-1 cells	–	–	Overexpression inhibited BCa cell proliferation, migration, and invasion	(70)
CircACVR2A (hsa_circ_0001073)	140 BCa patient tissues and matched NATs	T24, UM-UC-3, RT4, J82, 5637, HT-1376, TCCSUP BCa cell lines, and SV-HUC-1 cells	miR-626/EYA4 axis	–	Overexpression inhibited BCa cells proliferation, migration, and invasion *in vitro* and tumor progression and metastasis *in vivo*	(71)
Hsa_circ_0002024	20 BCa patient tissues and matched NATs	EJ, 5637, T24, and UMUC-2 and normal human urothelial cells	miR-197-3p	–	Upregulation suppressed BCa cells proliferation, migration, and invasion	(72)
Circ-FOXO3	49 BCa patient tissues and matched NATs	EJ and T24 BCa cell lines	miR-9-5p/TGFBR2 axis	–	Upregulation suppressed BCa cells proliferation, migration, and invasion	(73)
	30 BCa patient tissues and matched NATs	T24, UM-UC-3 and J82 BCa cell lines, and SV-HUC-1 cells	miR-191-5p	–	Overexpression increased apoptosis	(74)
CircNR3C1 (Hsa_circ_0001543)	42 BCa patient tissues and matched NATs	T24, EJ, UMUC3, J82, 5637 BCa cell lines, and SV-HUC-1 cells	-miR-23a-3p -miR-27a-3p/cyclin D1 axis	–	Upregulation suppressed BCa cells proliferation and progression *in vitro* and *in vivo via* arrest in the G0/G1 phase	(75)
CircMTO1	117 BCa patient tissues and matched NATs	UMUC3, SVHUC1, T24, J82, and 5637 and CCC-HB-2 cells	miR-221	–	Overexpression inhibited BCa cell migration and invasion *in vitro* and progression *in vivo*	(76)
Circ-ITCH	72 BCa patient tissues and matched NATs	5637, T24, J82, EJ, UMUC, TCC, 253J, and RT4 BCa cell lines and SV-HUC cells	-miR-224 -miR-17/p21 and PTEN axis	–	Downregulated circ-ITCH inhibited BCa cell proliferation, migration, and invasion *in vitro via* induction of the G1/S phase arrest Tumor progression was suppressed *in vivo*	(77)
CircST6GALNAC6	30 BCa patient tissues and matched NATs	T24, J82, UM-UC-3, 5637, and SW780 and SV-HUC-1 cells	miR-200a-3p/STMN1 signaling axis	–	Overexpression suppressed BCa cell proliferation and migration *in vitro* and metastasis *in vivo*	(78)
Circ_0071662	97 BCa patient tissues and matched NATs	BIU-87, T-24, EJ-28, and J82 BCa cell lines and SV-HUC-1 cells	miR-146-3p	–	Overexpression inhibited BCa cell proliferation and invasion	(79)
Hsa_circ_0018069	41 BCa patient tissues and matched NATs	T24, and Biu-87 BCa cell lines and SV-HUC-1 cells	miR-23c, miR-34a-5p, miR-181b-5p, miR-454-3p, and miR-3666	–	Downregulation correlated with more severe clinicopathological features	(80)
CircPICALM	168 BCa patient tissues and 40 NATs	T24, UM-UC-3, J82, and RT-4 BCa cell lines and SV-HUC-1 cells	miR-1265/STEAP4/pFAK-Y397 axis	–	Overexpression suppressed BCa cell invasion *in vitro* and metastasis *in vivo*	(81)
Hsa_circ_0091017	40 BCa patient tissues and corresponding NATs	5637, EJ, T24, UMUC-3, RT4 BCa cell lines, and SV-HUC-1 cells	miR-589-5p	–	Overexpression suppressed BCa cell proliferation, migration, and invasion	(82)
Circ-*ZKSCAN1*	68 BCa patient tissues and matched NATs	T24, UM-UC-3, 5637, and EJ BCa cell lines and SV-HUC-1 cells	miR-1178-3p/p21 axis		Overexpression suppressed BCa cell proliferation, migration, and invasion *in vitro* and also tumor progression and invasion *in vivo*	(83)
CircHIPK3	44 BCa patient tissues and matched NATs	T24T and UMUC BCa cell lines and SV-HUC-1 and HUVEC normal bladder cells	miR-558/HPSE axis	Angiogenesis	Overexpression repressed BCa cell migration and invasion *in vitro* and also tumor progression, metastasis, and angiogenesis *in vivo*	(84)
CircFAM114A2	31 BCa patient tissues and matched NATs	T24, J82, 5637, and 293T BCa cell lines and SV-HUC-1 cells	miR-762/△NP63 axis	–	Overexpression suppressed BCa cell proliferation, migration, and invasion *in vitro* and tumor growth *in vivo*	(85)
CircPTPRA	104 BCa patient tissues and matched NATs	T24 and UM-UC-3 BCa cell lines and SV-HUC-1 and HEK-293T cells	miR-636/KLF9 axis	–	Overexpression suppressed BCa cell proliferation and knockdown promoted it *in vitro* and tumor growth *in vivo*	(86)
CiRs-6	45 BCa patient tissues and matched NATs	T24 and UM-UC-3 BCa cell lines	miR-653/March1 axis	–	Overexpression inhibited BCa cell proliferation *in vitro* and tumor growth *in vivo*	(87)
CircFNDC3B	82 BCa patient tissues and 56 matched NATs	T24 and UM-UC-3 BCa cell lines and SV-HUC-1 cells	miR-1178-3p/G3BP2 axis	–	Overexpression repressed BCa cell proliferation, migration, and invasion *in vitro* and inhibited tumor growth and metastasis *in vivo*	(88)
CircUBXN7 (hsa_circ_0001380)	30 BCa patient tissues and matched NATs	T24, J82, EJ, RT4, and UM-UC-3 BCa cell lines and SV-HUC-1 cells	miR-1247-3p/B4GALT3 axis	–	Downregulation correlated with more severe clinicopathological features in BCa patients Overexpression suppressed BCa cell proliferation, migration, and invasion *in vitro* and tumor progression *in vivo*	(89)
CircCDYL	30 BCa patient tissues and matched NATs	EJ and T24T BCa cell lines and SV-HUC-1 cells	c-Myc	–	Overexpression repressed BCa cell proliferation and migration *in vitro*	(90)
Circ5912	45 BCa patient tissues and matched NATs	T24 and SW780 BCa cell lines	–	TGFβ	Knockdown increased BCa cell proliferation and invasion *in vitro* Overexpression decreased EMT through suppression of TGF-β2	(91)
CircRBPMS	90 BCa patient tissues and matched NATs	RT4, UM-UC-3, T24, 5637, and J82 BCa cell lines and SV-HUC-1 cells	miR-330-3p/RAI2/EMT-ERK axis	KRAS/ERK	Overexpression suppressed BCa cell proliferation and invasion *in vitro* and tumor progression and metastasis *in vivo*	(92)
CircLPAR1 (hsa_circ_0087960)	68 BCa patient tissues and matched NATs	5637 and T24 BCa cell lines and 293 cells	miR-762		Knockdown increased BCa cell invasion	(93)
CircRNA_000285 (hsa_circ_0000285)	146 BCa patient tissues and 98 matched NATs	HTB-9, T24, J82, SW780, and RT4 BCa cell lines and CCC-HB-2 normal bladder cells	–	–	Lower level was seen in chemoresistance to cisplatin	(94)
Cdr1as	32 BCa patient tissues and matched NATs	TCCSUP, 5367, T24, and EJ BCa cell lines	miR-1270/APAF1 axis	–	Cdr1as improved BCa cell’s chemosensitivity to cisplatin *in vitro* and *in vivo* Overexpression increased apoptosis in BCa cells	(95)

### Diagnostic and Prognostic Values of CircRNA in BCa

As explained above, dysregulated circRNAs cause disturbance in the cellular proliferation leading to malignancies, particularly BCa. On the other side, in the majority of the studies, dysregulation in circRNA expression has been statistically correlated with unfavorable clinicopathological features including high tumor size, histological grade, pathological stage, and presence of distant or lymph node metastasis in uni- or multivariate analyses in BCa patients. Therefore, as a consequence, it has been found that a dysregulation in the circRNA level can predict poorer survival (in terms of overall survival (OS), recurrence-free survival (RFS), disease-free survival (DFS), or progression-free survival (PFS)) and worse prognosis in Kaplan–Meier analyses.

As an example, it was formerly stated that hsa_circRNA_403658 is upregulated in BCa tissues compared to adjacent tissues (17). Kaplan–Meier analysis for the evaluation of survival showed that a high level of hsa_circRNA_403658 expression correlates with shorter survival in BCa patients. Implementation of the χ^2^ test to assess the association between circRNA expression and clinicopathological features has revealed a positive correlation between high hsa_circRNA_403658 expression and malignant characteristics including higher tumor volume (size ≥3 cm related to <3 cm), metastasis to distant places and advanced TNM stage (III–IV). In the univariate and multivariate Cox regression test for assessment of prognostic factors, it was demonstrated that hsa_circRNA_403658 is an independent factor for prediction of prognosis in BCa patients (17).

In another study (70), downregulated circRNAs hsa_circ_0077837 and hsa_circ_0004826 in BCa were found to be significantly associated with worse OS and RFS in BCa patients in Kaplan–Meier analysis. Univariate and multivariate Cox regression analyses confirmed that both circRNAs can act as independent prognostic factors compared to other factors in BCa patients. The area under the curve (AUC) for assessment of prognostic power of circRNAs revealed 0.775 and 0.790 values for hsa_circ_0077837 and hsa_circ_0004826, respectively, showing acceptable measures and suggesting their potentials as reliable biomarkers for prediction of worse prognosis in BCa patients.

Among other circRNAs, circASXL1 has been reported to have sensitivity and specificity of 0.686 and 0.769, respectively, which suggests its reliable diagnostic power in distinguishing the BCa patients from healthy people (38). A number of circRNAs whose prognostic or diagnostic values have been studied in BCa are shown in **Table 3**.

**Table 3:** An overview to the diagnostic and prognostic values of dysregulated circRNAs in BCa.

Description	Area under curve	Sensitivity	Specificity	Kaplan–Meier analysis	Univariate Cox regression	Multivariate Cox regression	Other correlation tests	Ref. (s)
Hsa_circRNA_403658 upregulation in BCa patients	–	–	–	hsa_circRNA_403658 high expression correlated with shorter survival in BCa patients.	Increased hsa_circRNA_403658 level was correlated with advanced clinicopathological features such as increased tumor size(≥3 cm), metastasis to distant places, and malignant TNM stage(III–IV).	Increased hsa_circRNA_403658 level was correlated with advanced clinicopathological features including larger tumor size(≥3 cm), lymph node metastasis, distant metastasis, and malignant TNM stage (III-IV).	χ^2^ test showed a positive correlation between high hsa_circRNA_403658 expression and malignant clinicopathological features including larger tumor size, advanced tumor TNM stage, and distant metastasis.	(17)
					hsa_circRNA_403658 can act as an independent prognostic factor for BCa patients			
Hsa_circ_0003221 (circPTK2) upregulation in tissue and blood samples of BCa patients	–	–	–	–	–	–	Student’s *t* test showed that the expression level is correlated with tumor size, lymph node metastasis, and T stage at BCa patients (p < 0.05)	(30)
Hsa_circ_0072995 (circRGNEF) upregulation in BCa patients	–	–	–	–	–	–	High expression level was positively correlated with lymph node metastasis, high T stage, and advanced grades of BCa and also associated with worse prognosis.	(33)
CircGprc5a upregulation in BCa patients	–	—	–	High circGprc5a expression correlated with poorer survival and prognosis in BCa patients.	–	–	–	(34)
CircZFR upregulation in BCa patients	0.8216	–	–	Higher circZFR correlated with poorer prognosis and survival in BCa patients.	circZFR expression correlated with worse PFS and OS.		The chi-square tests showed correlation between circZFR expression and tumor stage, grade, lymph node metastasis, and recurrence in BCa patients.	(37)
CircASXL1 upregulation in BCa patients	0.770	0.686	0.769	BCa patients with higher levels of circASXL1 levels had poorer OS.	High circASXL1 expression correlated with more severe clinicopathological features including higher tumor grade, pathological T stage, distant, and lymph node metastasis.	High circASXL1 expression correlated with malignant clinicopathological features including advanced pathological T stage, distant and lymph node metastasis.	–	(38)
Hsa_circ_0001944 upregulation in BCa patients	–	–	–	High Hsa_circ_0001944 expression correlated with worse prognosis in BCa patients.	–	–	The Pearson’s correlation test showed correlation between hsa_circ_0001944 higher expression and larger tumor size, advanced T stage, higher grade, and lymph node metastasis in BCa patientsAlso, poorer prognosis was predicted for patients with higher hsa_circ_0001944 levels.	(47)
CircPRMT5 upregulation in BCa patients	–	–	–	BCa patients with high circPRMT5 or miR-30c expression exhibited poorer survival rate. Correlated with worse prognosis.	–	–	The x^2^ test showed that circPRMT5 serum and urine levels in BCa patients are associated with metastasis.	(48)
Hsa_circ_0041103 upregulation in BCa patients	–	–	–	Hsa_circ_0041103 expression correlated with unfavorable OS in BCa patients.	–	–	The two-paired independent *t*-test demonstrated positive correlation between high levels of hsa_circ_0041103 and advanced clinicopathological features including larger tumor size, higher pathological T stage, and lymph node metastasis in BCa patients.	(50)
cTFRC upregulation in BCa patients	–	–	–	Patients with higher cTFRC expression level had poorer OS.	–	–	cTFRC expression was positively correlated with advanced tumor T stage, higher grade and lymphatic invasion at BCa patientscTFRC was associated with poor prognosis.	(96)
Circ_0061140 upregulation in BCa patients	–	–	–	BCa patients with high circ_0061140 expression exhibited worse prognosis compared to those with lower levels.	–	–	The *χ* ^2^ test showed correlation between high circ_0061140 levels and lymph node or distant metastasis.	(57)
Circ_0067934 upregulation in BCa patients	–	–	–	BCa patients with high circ_0067934 level had shorter 5-year OS and disease-free survival.	–	–	The χ^2^ test showed a positive correlation between high circ_0067934 levels and advanced clinicopathological features including tumor size, higher pathological stage, and lymph node metastasis.	(59)
Circ-BPTF upregulation in BCa patients	–	–	–	Patients with higher circ-BPTF expression level had worse OS.	–	–	High cTFRC expression was positively correlated with advanced tumor stage and recurrence in BCa patients.	(64)
CircFUT8 downregulation in BCa patients	–	–	–	Lower circFUT8 levels in BCa patients correlated with worse OS and poor prognosis.	–	–	The chi-square test demonstrated an association between low circFUT8 expression and worse clinicopathological features including lymph node metastasis and high histological grade.	(68)
Hsa_circ_0077837 and Hsa_circ_0004826 downregulation in BCa patients (low: 35 for both and high: 35 for both)	0.775 and 0.790 for hsa_circ_0077837 and hsa_circ_0004826, respectively	–	–	Downregulated hsa_circ_0077837 and hsa_circ_0004826 were associated with lower OS and RFS in BCa patients.	Correlation between high expression of both circRNAs, tumor stage, grade, and lymph node metastasis with shorter OS and RFS in BCa patients.	Correlation between high expression of both circRNAs and lymph node metastasis with shorter OS, and also high expression of hsa_circ_0077837 and lymph node metastasis with shorter RFS in BCa patients.	–	(70)
					Both circRNAs can act as independent factors for prediction of prognosis in BCa patients.			
CircACVR2A downregulation in BCa patients	–	–	–	Low circ-BPTF expression level was associated with worse OS and prognosis in BCa patients.	–	–	Chi-square test showed correlation between low circACVR2A expression and advanced tumor stage, grade, and lymph node metastasis in BCa patients.	(71)
Circ-ITCH downregulation in BCa patients	–	–	–	Lower circ-ITCH level positively correlated with shorter OS and poorer prognosis in BCa patients.	–	–	Downregulated circ-ITCH was significantly associated with high pathological tumor stage in BCa patients.	(77)
Circ_0071662 downregulation in BCa patients	–	–	–	Circ_0071662 expression was positively correlated with survival rate in BCa patients.	–	–	Low Circ_0071662 expression correlated with lymph node and distal metastasis and poorer prognosis in BCa patients.	(79)
Hsa_circ_018069 downregulation in BCa patients (diminished in 80.5% (33/41) of cases)	0.709	0.976	0.463	–	–	–	The Student’s *t*-test showed that hsa_circ_018069 downregulation correlated with more severe clinicopathological features including high tumor grade, pathological T stage, and tumor muscular invasion.	(80)
CircPICALM downregulation in BCa patients	–	–	–	BCa patients with a diminished level of circPICALM exhibited poorer OS related to those with high levels.	circPICALM expression, histological grade, pathological T stage, and lymph node metastasis correlated with survival in BCa patients.	circPICALM expression and lymph node metastasis demonstrated as independent features for prediction of prognosis in BCa patients.	The chi-square test showed that circPICALM downregulation correlated with unfavorable clinicopathological features including high histological grade, pathological T stage, and lymph node metastasis in BCa patients.	(81)
Circ-ZKSCAN1 downregulation in BCa patients	–	–	–	Downregulated circ-ZKSCAN1 correlated with worse OS and disease-free survival and predict poorer prognosis for BCa patients.	–	–	The chi-square test demonstrated an association between low circ-ZKSCAN1 levels and advanced clinicopathological features including high histological grade, pathological T stage, and lymph node metastasis in BCa patients.	(83)
CiRs-6 downregulation in BCa patients	–	–	–	Higher ciRs-6 was positively correlated with good OS in BCa patients.	–	–	One-way ANOVA test showed that higher ciRs-6 expression was associated with lower tumor grade, pathological T stage, and better prognosis in BCa patients.	(87)
Hsa_circ_0018069 downregulation in BCa patients	0.709	0.976	0.463	–	–	–	The Student’s *t*-test showed correlation between low hsa_circ_0018069 levels and clinicopathological features including higher tumor grade, advanced T stage, and muscular invasion depth in BCa patients.	(80)
CircUBXN7 downregulation in BCa patients	–	–	–	Patients with decreased circUBXN7 levels showed shorter OS.	–	–	The chi-square analysis showed correlation between low circUBXN7 expression and advanced pathological T stage and more severe grades in BCa patients.	(89)
Circ5912 downregulation in BCa patients	–	–	–	Patients with higher circ5912 expression had longer OS compared to those with lower levels.	–	–	The one-way ANOVA test showed correlation between higher levels of circ5912 and favorable clinicopathological features including lower tumor grade, stage, and metastasis in BCa patients.	(91)
CircFNDC3B downregulation in BCa patients	–	–	–	Patients with lower circFNDC3B levels showed decreased survival.	–	–	The chi-square test revealed a positive correlation between lower circFNDC3B levels and highly advanced clinicopathological features such as higher histological grade, T stage, and metastasis to lymph nodes.	(88)
Circ-ITCH downregulation in BCa patients	–	–	–	Low circ-ITCH levels in BCa patients were positively correlated with poorer OS.	–	–	The chi-square test showed a correlation between low circ-ITCH expression level and more advanced tumor grade in BCa patients.	(77)
Hsa_circ_0000285 downregulation in BCa patients				Higher hsa_circ_0000285 expression was associated with longer OS in BCa patients.	hsa_circ_0000285 level correlated with prognosis in BCa patients.	hsa_circ_0000285 level is an independent prognostic factor for BCa patients.	The chi-square demonstrated correlation between hsa_circ_0000285 expression and clinicopathological features.	(94)
CircLPAR1 downregulation in BCa patients	–	–	–	BCa patients with low circLPAR1 levels had decreased survival compared to those with higher levels.	A correlation was seen between low circLPAR1 levels and decreased DSS in BCa patients.		–	(93)

OS, overall survival; RFS, recurrence-free survival; DDS, disease-specific survival.

## Discussion

Circular RNAs (circRNAs) are covalently closed nucleic acid strands which are classified as non-coding RNAs and mainly do not code any protein. They have been found to play a role in gene regulation in several stages. CircRNAs show cell-, tissue-, or species-specific tropism and are known to be dysregulated in tissues in a number of cancers. They have been found to either act as tumor suppressors or exhibit oncogenic roles on overexpression. The causative mechanisms of circRNAs’ role in tumorigenicity are vastly being studied. Their dysregulation has mainly been associated with disturbances in cell cycle regulation through activation of several signaling pathways.

In this review, we summarized a number of studies conducted on dysregulated circRNAs in BCa. Dysregulation includes any increase or decrease in circRNA expression levels in BCa tissues or cell studies compared to normal adjacent tissues. To assess the circRNA dysregulation, some high-throughput technologies like RNA sequencing and confirmatory qRT-PCR have been employed. Upregulated circRNAs in the first section (**Table 1**) have been found to accelerate tumor cell proliferation and enhance migration and invasion of cancer cell *in vitro*. *In vivo* studies have shown increased tumor progression when these circRNAs are overexpressed. Suppression of upregulated circRNAs *via* specific siRNAs in BCa has confirmed repression of proliferative and migratory potential following their silencing.

Downregulated circRNAs (**Table 2**), on the other hand, have been known to exert their anti-tumorigenic roles *via* suppression of tumor cell proliferation, migration, invasion, and metastasis. Overexpression experiments have confirmed tumor-suppressing roles of the second class of circRNAs *via* diminishing tumor cell malignant behaviors.

Furthermore, correlation studies have shown a significant association between dysregulated circRNA expression levels and worse clinicopathological features including higher tumor size, distant or lymph node metastasis, higher histological grade, pathological stage, and advanced TNM stage in BCa patients (**Table 3**). Statistical analyses have also demonstrated that dysregulation of circRNAs can be used as independent prognostic factors for BCa patients. Acceptable AUC, specificity, or sensitivity values in diagnostic analyses have revealed the diagnostic power of circRNAs in distinguishing BCa from other diseases. Taken together, circRNAs have been suggested as markers with reliable prognostic and diagnostic potential which can be used as biomarkers in either diagnosis or prediction of prognosis in BCa patients. Not only circRNAs but also other ncRNAs like lncRNAs have shown high stability in biological fluids, making them good biomarkers with easy detection for a number of human diseases particularly diverse types of cancers including BCa, breast cancer, hepatocellular carcinoma, and colorectal cancer (97, 98). Exosomes as extracellular vesicles involved in cellular communications are particularly shown to contain circRNAs in high concentrations and in a stabile form (99). These membranes can be beneficial in the detection of malignancies when derived from cancer cells spreading to blood and detectable in serum. Newly identified circRNAs have been introduced through high-throughput approaches such as next-generation sequencing (NGS), and microarray analysis, which make the huge identification of ncRNAs possible, while qRT-PCR is the main technique with potential in clinical diagnosis and quantification of circRNAs for both prognostic and diagnostic goals, and it is also used as the confirmatory method upon a novel ncRNA as previously reported (100). Vast application of qRT-PCR in quantification of a circRNA, however, is possible when the junction/fusion site is identified (8). Moreover, other technological drawbacks require to be addressed for clinical applications of circRNAs.

*In vitro* and xenograft studies have confirmed the suitability of circRNAs as therapeutic targets in cancers. However, several issues such as biosafety ones should be solved before application of circRNA-targeting methods in clinical settings.

Taken together, although circRNAs have shown roles in the development and progression of various human cancers, and their quantification have demonstrated excellent potential in distinguishing patients with BCa from healthy individuals, it seems that application of circRNAs as novel biomarkers needs further investigations, more time, and addressing of technological problems to enter in clinical settings.

## Author Contributions

SGF and SN wrote the draft and revised it. MT designed and supervised the study. BMH, HJH, FR, and AB collected the data and designed the figures and tables. All authors contributed to the article and approved the submitted version.

## Conflict of Interest

The authors declare that the research was conducted in the absence of any commercial or financial relationships that could be construed as a potential conflict of interest.

## Publisher’s Note

All claims expressed in this article are solely those of the authors and do not necessarily represent those of their affiliated organizations, or those of the publisher, the editors and the reviewers. Any product that may be evaluated in this article, or claim that may be made by its manufacturer, is not guaranteed or endorsed by the publisher.
